# Bridging Conservation Gaps: Evaluating Habitat Mapping Methods for Alpine River Ecosystems

**DOI:** 10.1007/s00267-026-02527-9

**Published:** 2026-06-21

**Authors:** Wiebke Winkelhues, Thomas C. Wagner, Helmut Kudrnovsky, Carmen Rethschulte, Michael Reich

**Affiliations:** 1 Institute of Environmental Planning, Leibniz University of Hanover, Hannover, Germany; 2 School of Life Sciences, Technische Universität München, Freising-Weihenstephan, Germany; 3 University of Vienna, Wien, Austria

**Keywords:** Dynamic habitats, Habitats directive, Natura 2000, Alpine braided rivers, Habitat suitability model, Myricaria germanica

## Abstract

Natural habitats are globally threatened and fragmented, posing challenges for dynamic ecosystems dependent on ecological processes and intact habitat networks. Our study, therefore, examines the implementation of the European Union’s Habitats Directive in safeguarding the habitat type “Alpine rivers with their ligneous vegetation with *Myricaria germanica*”. Using Bavarian and Austrian mapping guidelines, alongside simple and habitat suitability models, we identified disparities and limitations in current habitat delineation methods. Our findings reveal substantial inconsistencies between the Bavarian and Austrian mapping guidelines and the species’ potential habitat predicted by the habitat suitability model. The Bavarian method overestimates the extent of the habitat type by also classifying areas in advanced successional stages as such, covering 88% of the study area. Habitat suitability modeling shows this exceeds the suitable habitat by up to 74%. Compared to the more selective Austrian method, the Bavarian method maps 14 times more area. The Austrian method focuses on the current habitat occupancy of *M. germanica*, a crucial factor in detecting changes in distribution and habitat quality in dynamic river systems. However, monitoring pioneer, pre- and sub-successional habitats alongside existing populations is essential to fully protect metapopulation dynamics. Habitat Suitability Modeling offers an opportunity to complement field surveys for this purpose. Our findings further highlight the need for more consistent cross-border mapping and standardized assessment criteria to accurately track habitat changes, supporting effective implementation of instruments such as the Nature Restoration Regulation and ensuring long-term conservation and restoration of dynamic ecosystems.

## Introduction

In recent decades, natural and semi-natural habitats have declined dramatically worldwide (Newbold et al. 2015, Jacobsen et al. 2019) driven by factors such as agricultural intensification, and climate change (Bayer & Manica 2020, Leadley et al. 2010). As a result, remaining habitat are often fragmented and in deteriorating condition (e.g., Haddad et al. 2015, Araújo et al. 2011). In response, governments worldwide have implemented conservation and restoration policies (e.g., CBD, Aichi Biodiversity Targets, European Commission 1992, 2024). Their effective implementation requires robust methods to map distribution, asses condition, and detect structural changes of habitats. Since the Bern Convention in 1979, later implemented by the Habitats Directive (Council of Europe 1979, European Commission 1992), the EU has adopted directives that directly operationalize global biodiversity goals, such as those outlined in the Convention on Biological Diversity (CBD). This makes the EU a reference point for global conservation policy (Epstein et al. 2015). The Habitats Directive is particularly noteworthy for its legally binding obligations, which have recently been reinforced by the Nature Restoration Regulation (NRR, European Commission 2024), requiring EU member states to maintain or restore species and habitats to a favorable conservation status (European Commission 1992). Additionally, the Natura 2000 network, the world’s largest system of protected areas, exemplifies large-scale habitat protection and connectivity, serving as a model for similar efforts worldwide (EEA 2020).

In the Habitats Directive, more than 200 habitat types have been identified as particularly worthy of protection (European Commission 1992). To conserve these habitats, protected areas must be designated to maintain or restore a good conservation status and thus achieve the objectives set by the Habitats Directive. The “conservation status” is defined as “the sum of influences […] that may affect its long-term natural distribution, structure and functions as well as long-term survival of its typical species” (European Commission 1992). European Member States must regularly monitor and report the conservation status of these habitat types to the European Commission (European Commission 1992, Evans & Arvela 2011). For the evaluation on the local level, habitat patches must be mapped in the field. The mapping of these habitats is primarily based on the presence of specific key species and the ecological characteristics of the habitat (European Commission 2013). Additionally, for each mapped habitat, further criteria must be evaluated to assess the “degree of conservation” of the respective habitat. This should consider structural aspects of the habitat, complementary species, and impairments (DG Environment 2023). The Habitats Directive provides a general framework for these assessment criteria. However, Member States are responsible for implementing these criteria and may adjust, provided they are based on the interpretation manual (European Commission 2013, DG Environment 2023). This allows regional differences to be considered, but it also means that the same situation may be assessed differently.

Such differences in assessment are particularly prevalent in highly dynamic and frequently disturbed ecosystems such as alpine rivers or dune ecosystems, as the habitat situation and configuration are generally subject to spatio-temporal variations (Sitzia et al. 2021, European Commission 2013, Doody 2013). Within such dynamic ecosystems, certain species exist as a metapopulation, distributed over many suitable habitat patches that exist for a limited time (Leibold et al. 2004, Holyoak et al. 2020). The extinction of populations, e.g., due to the loss of habitat patches following the disturbance characteristic for the system (e.g., flooding), is compensated for by the colonization of newly created, previously unoccupied, or once lost habitat patches (Levins 1969, Hanski 1998). Such species depend on the disturbance processes in the underlying ecosystem.

One such metapopulation forming species characteristic for alpine rivers, is *Myricaria germanica*. It is protected under the habitat type “Alpine rivers with their ligneous vegetation with *M. germanica*”. According to the European Commission (2013), this habitat type is classified as *Salici-Myricarietum*, an association of *M. germanica* and various willows (*Salix ssp*.) found on gravel deposits rich in fine silt, characteristic in braided river sections with disturbance frequencies of five to ten years (Kalníkova et al. 2021, Moor 1958, Müller 1995). In the active floodplain, *M. germanica* rarely exceeds 30 years of age and exhibits significant population fluctuations (Kudrnovsky & Höbinger 2015, Wagner & Wöllner 2024). Thus, *M. germanica* and the related habitat type “Alpine rivers with their ligneous vegetation with *M. germanica”* serve as ideal examples of metapopulations and dynamic habitats (Werth & Scheidegger 2014, Kudrnovsky & Höbinger 2015).

The area currently occupied by species that form metapopulations, such as *M. germanica*, does not reflect the extent of the currently available suitable habitat. Particularly with dispersal-limited plants, the number of propagules reaching a newly created habitat is often low (Wagner & Wöllner 2023). Furthermore, time is required for founder organisms to spread and develop into new populations capable of colonizing further new habitats. Using the presence of a species to identify suitable habitats is, therefore, misleading in dynamic environments. In some circumstances where the predicting factors are available on a spatial scale, habitat suitability models (HSM) can be used to determine the actual patches of suitable habitat available. This not only allows for a more precise delineation of the species’ potential habitat, but also enables the determination of the fragmentation of its habitat area, a factor relevant for the successful maintenance of a metapopulation (Wagner & Wöllner 2024).

As the conservation of dynamic habitats such as “Alpine rivers with their ligneous vegetation with *M. germanica*” requires protection of the entire ecosystem and its dynamic processes (Wohl et al., 2005; Suding et al., 2016), monitoring must also consider these factors. Since metapopulations and thus also dynamic habitats respond to impairment of habitats non-linearly and with a time lag, this becomes particularly important (Hanski 1996, Andren 1995). Due to its legally binding nature, the Habitats Directive is one of the most effective instruments for nature conservation in Europe. Utilizing the habitat type “Alpine rivers with their ligneous vegetation with *M. germanica*” as a case study, our objective was to evaluate the efficacy of national mapping guidelines in translating the conservation objectives of the Habitats Directive into practice for highly dynamic ecosystems, thereby supporting informed and appropriate management decisions. To achieve this aim, we addressed the following research questions:

1. How do the habitat mapping guidelines of two member states (Germany, Bavaria and Austria) compare in delineating the habitat type “Alpine rivers with their ligneous vegetation with *M. germanica”*?
2. How is the habitat situation of *M. germanica* assessed using intuitive assessment and habitat suitability modeling?
3. How do the habitat delineations resulting from the Member States’ mapping guidelines compare with those derived from intuitive assessment and habitat suitability modeling?
4. In what circumstances can alternative approaches, such as intuitive assessments and habitat suitability modeling, effectively complement or enhance the monitoring of dynamic habitats under the Habitats Directive?

## Methods

### Study Area

Our study was conducted along the Isar River in the Bavarian Alps. The Upper Isar, between Mittenwald and the Sylvenstein reservoir, is the last remaining near-natural alpine river section in Germany and one of the few remaining in the Northern Alps (Maier et al. 2021, Reich 2006). Although hydrology, as well as the morphology of this stretch, have been affected by water abstraction and bedload deficiency, it still has a wide braided floodplain with a largely natural braiding characteristic covering the typical habitat mosaic of alpine rivers. The stretch is of high conservation value and hosts several endangered plant and animal species. Accordingly, the last major population of *M. germanica* (habitat type 3230), as well as other typical braided river habitats, such as pioneer vegetation of herbaceous vegetation (“Alpine rivers and the herbaceous vegetation along their banks”, 3220) and lavender willow (*Salix eleagnos*) bushes (“Alpine rivers and their ligneous vegetation with *Salix eleagnos*”, 3240), can be found along this river stretch (EEA 2023, EEA 2016, European Commission 2013).

**Fig. 1:**
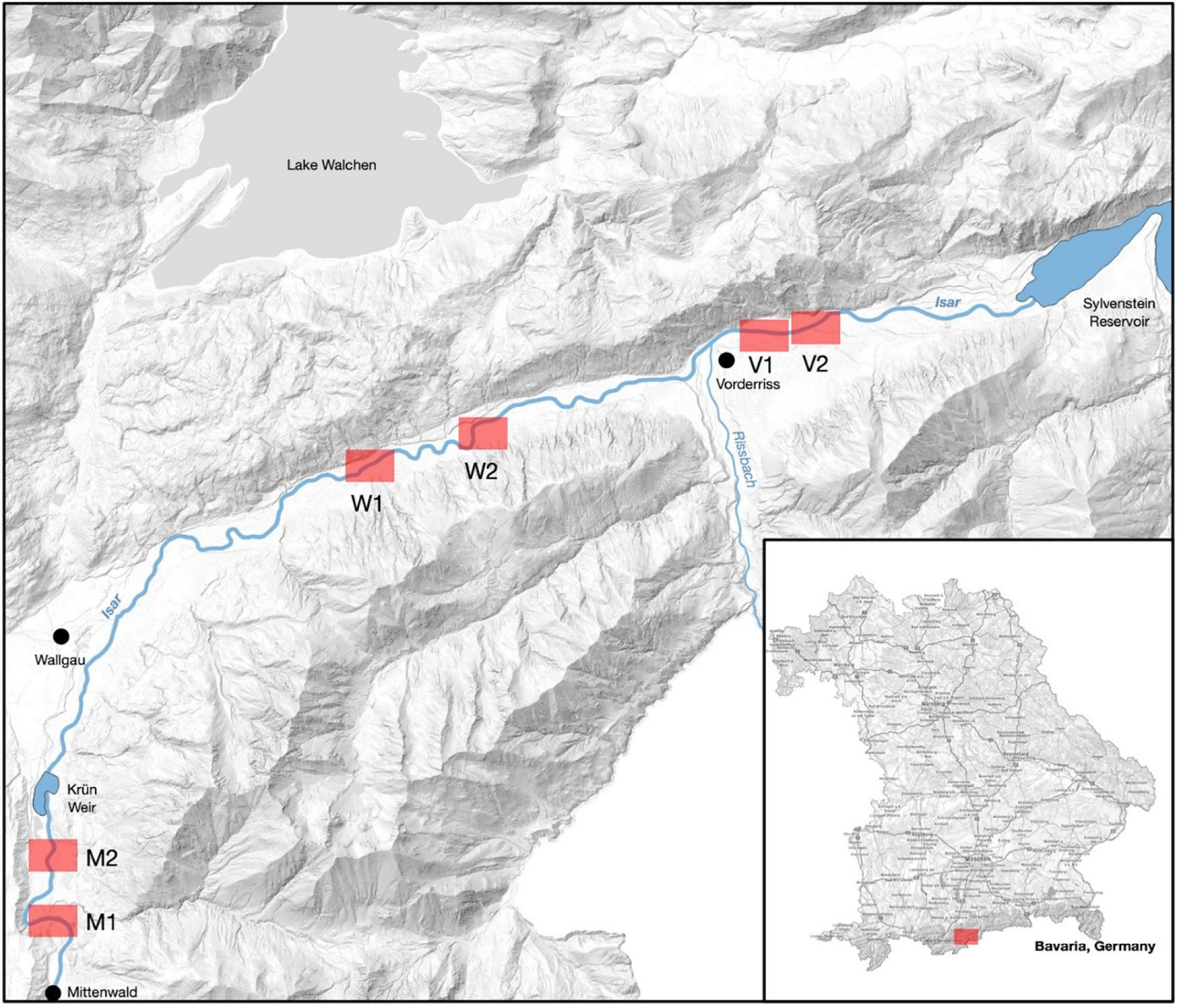
Overview map of the study region in Bavaria, Germany, and the sample areas along the Upper Isar River. M1, M2: sample areas with natural flood and morphology dynamics near Mittenwald, W1, W2: sample areas in the near-natural river section, natural flood and morpholgy dynamics impacted by the Krüner weir between Wallgau and Vorderriss, V1, V2: sample areas in the most natural (reference) section between Vorderriss and the Sylvenstein reservoir

We selected two sample areas within each of three differently degraded river sections located in Germany (Fig. 1), to evaluate the performance of the Bavarian and Austrian mapping approaches under different impairment conditions (Supplementary material, S1). We also assessed changes in habitat conditions along the river using a simple, intuitive delineation approach and a habitat suitability model.

The first river section is located at the Isarhorn, downstream of Mittenwald (Fig. 1, M1, M2). Although the Isar has been straightened upstream for flood protection of the town of Mittenwald, natural flood and morphology dynamics are maintained in this river section (Wagner et al. 2024). In the second river section between Wallgau and Vorderriß, the Isar is widely branched (Reich 2006). Here, the Krüner weir diverts up to 25 m3/s over the Obernach Channel towards Lake Walchen, leaving a minimum residual flow of 3–4.8 m3/s (Winter, Summer; LfU 2023). This reduces flow velocity, transport capacity, bedload, and flood peaks. This favors the spread of more competitive willow species at later stages of succession on sites with less river dynamics, stabilizing gravel banks, and river beds (Reich 2006, Juszyk et al. 2020, Wagner et al. 2024). The third river section, between Vorderriß and the Sylvenstein reservoir, is characterized by a large braided floodplain and near natural sediment dynamics (Wagner et al. 2024). Whilst also affected by altered discharge and bedload blockage at the Krüner weir, the bedload deficit is mainly compensated by the Rissbach, which transports significant bedload during floods (Maier et al. 2021).

Within each river section, two sample areas of a minimal length of 100 meters (LFU 2022a) were delineated. The scale was chosen to capture multiple habitat patches and reflect the local distribution of *M. germanica*, given that the dispersal of propagules along the river ranges from 10 to 100 meters (Sitzia et al., 2021). We selected sample areas where the presence of *M. germanica* was recently confirmed (Wöllner et al. 2019, Reich & Rethschulte 2021).

### Data Acquisition

#### Mapping of the habitat type “Alpine rivers with their ligneous vegetation with *M. germanica”* (3230)

To identify and delineate the habitat type, we conducted extensive field surveys from May to June of 2023. Following the Bavarian mapping guidelines (LWF & LfU 2018), we first mapped *M. germanica*, the key species for habitat identification, within the sample areas. If *M. germanica* was found in a sample area, the entire river section was designated as the habitat type (LWF & LfU 2018). Here, the presence of a single individual is sufficient to delineate an entire watercourse section, including all gravel bars, the watercourse as well as the riverside areas. Other habitat types listed in Annex I of the Habitats Directive, are delineated separately only if no individuals of *M. germanica* are present (LWF & LfU 2018, LfU 2022b). This applies, for example, to the habitat of “Hydrophilous tall herb fringe communities of plains of the montane to alpine levels” (habitat type 6430) and “Alluvial forests with *Alnus glutinosa* and *Fraxinus excelsior*” (habitat type 91E0*) (LWF & LfU 2018, European Commission 2013). Existing habitat maps from management plans (Bayerische Forstverwaltung 2017) served as a preliminary to identify adjacent habitat types. However, all delineations were verified and adjusted during fieldwork to account for recent successional or flood-related changes in the river corridor. Additionally, areas where *M. germanica* was absent, where vegetation cover in the shrub and tree layer exceeded 40%, and where no characteristic species occurred, were excluded from the demarcation (LWF & LFU 2018).

According to the Austrian requirements (Ellmauer et al. 2020), we only delineated gravel bank areas as habitat type if the *M. germanica* covered more than 1% of a habitat patch. Additionally, the Austrian method includes a threshold for the cover ratio of *M. germanica* and *Salix* species in the shrub layer. Accordingly, we only delineated habitat patches where the cover of *M. germanica* in the shrub layer was greater than that of grey alder (*Alnus incana)* and/or *Salix* species. Otherwise, the habitat patch was assigned to other alpine river habitat types (Ellmauer et al. 2020). Accordingly, this threshold is used to assign *M. germanica* stands to other habitat types as succession progresses.

#### UAV mapping

UAV flights were conducted using a commercial DJI drone equipped with an RGB camera during the peak of the growing season (June-August 2023). Flights were performed at 40 m above ground level, resulting in a ground resolution of approximately 2 cm/px. All flights took place under mean water level (MQ, gauging station “Rissbachdüker”; Bavarian Hydrological Service). The aerial images were processed using Agisoft MetaShape 2.1.2 (Wöllner & Wagner 2019) to generate a point cloud, as well as orthomosaics and a digital surface model (DSM). Ground points were automatically identified (max angle 10°, max distance 0.1 m, max terrain slope 90°, max cell size 10 m, erosion radium 0.02 m, any return) and used to generate the digital terrain model (DTM). The height accuracy of the DSM and DTM) is ±5 cm.

#### Simple model

As a simple model, we assessed the habitat suitability for germination and establishment of *M. germanica* based on vegetation cover (percent) and the surface sand content of the soil substrate (in percent), as it is often used by practitioners (Wagner & Wöllner 2024). Both variables can easily be estimated on-site or from aerial images, and their determination does not require to employ GIS analysis or even more sophisticated approaches. The delimitation was done by applying threshold values for the two variables, which are usually taken from the literature (e.g., Sitzia et al. 2021) or when only qualitative information is available (e.g., “grows on predominantly sandy sites”, “open to sparsely vegetated”; Philippi 1995), are sometimes set arbitrarily or according to personal experience. In our case, we used rounded thresholds derived from the 80% surface range envelope, i.e., the envelope defined by the values of our environmental variables within the 80% quantile of ~10.000 sampled individuals of *M. germanica* (Wagner & Wöllner 2024). Accordingly, a suitable habitat is defined as having a total vegetation cover of less than 40% and a sand content of the surface substrate of more than 25%.

#### Habitat suitability model

To determine as accurately as possible the habitat suitable for germination and establishment of *M. germanica*, we used a Habitat Suitability Model (HSM). We used *vegetation cover*, the average *height of the vegetation*, the *height above normal water level*, and the *proportion of sand* in the surface substrate as explanatory environmental factors. These parameters represent the most important site factors for *M. germanica* (Sitzia et al. 2021, Wagner & Wöllner 2024) and can explain about 95% of the species’ occurrence in corresponding models (Wagner & Wöllner 2024). The basis for determining these factors are orthomosaics derived from UAV flights conducted during the height of the growing season between Jun-Aug. Ground resolution is approximately 2 cm/px. The aerial images were processed using Agisoft MetaShape 2.1.2 (Wöllner & Wagner 2019) and converted into a point cloud that was used to generate orthomosaics and digital surface model (DSM). Ground points were automatically identified (max angle 10°, max distance 0.1 m, max terrain slope 90°, max cell size 10 m, erosion radium 0.02 m, any return) and used to generate the digital terrain model (DTM). Height accuracy of the DSM and DTM) is ±5 cm. Vegetation cover was determined by calculating the Excessive Green Vegetation Index (ExG) from the RGB images and then applying the Otsus threshold (i.e., ExG values above the threshold represent vegetation and, therefore, cover, see Wöllner & Wagner 2019). Vegetation height was calculated as (DSM - DTM)/vegetation cover. The division by vegetation cover ensures that only height values for pixels classified as vegetation are determined, i.e., dead wood or other non-vegetation structures result in NA values (“not available”) and are subsequently ignored.

To determine the terrain height above the normal water level (MQ, gauging station “Rissbachdüker”; Bavarian Hydrological Service), the water surface was first modeled using height-accurate reference points, and the surface thus represented was extrapolated to the entire area as a triangular irregular network (TIN). The height above MQ was then calculated as the difference between the DTM and the TIN.

The proportion of sand in the surface substrate was determined using a computer vision model based on tensorflow (for details, see Wagner & Wöllner 2024). All resulting raster with the explanatory variables were then resampled to a 5x5m resolution with the averages calculated.

The habitat suitability model was implemented in R (R Core Team 2024) with the *biomod2* package (Thuiller et al. 2024). A gradient-boosted regression (GBM) was used as a model variant, as it works well with background data and avoids overfitting (Wagner & Wöllner 2024). The model was tuned using the *biomod* tuning function. The model with the lowest root mean square error was chosen, resulting in a final model, with a shrinkage of 0.1, 7 interactions, a minimum of 5 objects in node and 100 trees with a bag fraction of 0.8.

For the model’s training, presence data for *M. germanica* in a 2 km long section directly downstream of the study site W2 were used. As the requirements for the establishment of juveniles significantly differ from the conditions tolerated by older plants (Wagner & Wöllner 2024), only juvenile individuals less than one year old were selected for training. To reduce spatial autocorrelation, the initial 2,764 juveniles were thinned to one individual per 5x5m grid cell, resulting in 885 presence points. These were supplemented by ten sets of 885 randomly generated pseudo-absences. To account for metapopulation dynamics, where suitable habitats remain unoccupied due to dispersal limitation or colonization lags (Drielsma & Love 2021, Wagner & Wöllner 2024), pseudo-absence selection was restricted to the active river corridor and locations outside the 95% surface range envelope (SRE). This ensures that pseudo-absences are drawn from environmentally unsuitable areas rather than merely unoccupied but suitable sites. A random fraction of 70% of the presence/absence data was used for training and 30% for model evaluation. The resulting models were evaluated using ROC and TSS, and their cut-off values and the relative importance of the predictor variables were determined. The model set was then projected onto the study sites, the results averaged, and the cut-off applied to produce binary raster with the predicted suitable habitat area. These raster were smoothed using a 3×3 focal filter function to remove regions with less than four neighboring cells. Finally, the results were validated against the census data in the respective study site.

#### Comparison of mapping and modeling results

The habitat suitability model could predict the potential habitat and the presence of *M. germanica* with high accuracy. The ROC of the average model was 0.97, and the TSS value 0.82 (S3, a). Validated against the actual presence data of *M. germanica*, the model correctly predicted 94% of all *M. germanica* individuals (S2). The most important variable was *vegetation cover* (57%), followed by *height above mean water level* (18%), *sand* (17%), and finally, *vegetation height* (7%), (S3, b).

The high accuracy of the habitat suitability model and the validation results confirm that the model provides a realistic representation of the habitat situation and identifies the habitat suitable for *M. germanica* with high accuracy. Thus, the habitat suitability model accurately represents the potential habitats for *M. germanica*. This allows the areas of the habitat type delineated by the Habitats Directive mapping approaches to be related to those predicted by the HSM. Falsely positive or negative predicted areas and the proportion of totally falsely attributed areas were determined. In this context, a false positive refers to areas that are identified as belonging to the habitat type by the Habitats Directive delineation, although the Habitat Suitability Model (HSM) indicates that they are not suitable for M.g. This may indicate that areas in advanced stages of succession - which no longer meet the defining characteristics of the habitat type - are still classified as such. In contrast, a false negative indicates that a potential habitat, which could be a vegetation-free gravel bar, is not designated as a habitat type under the Habitats Directive. Finally, general occupancy and the number of plant individuals lying outside the identified suitable habitat area were determined.

## Results

**Fig. 2:**
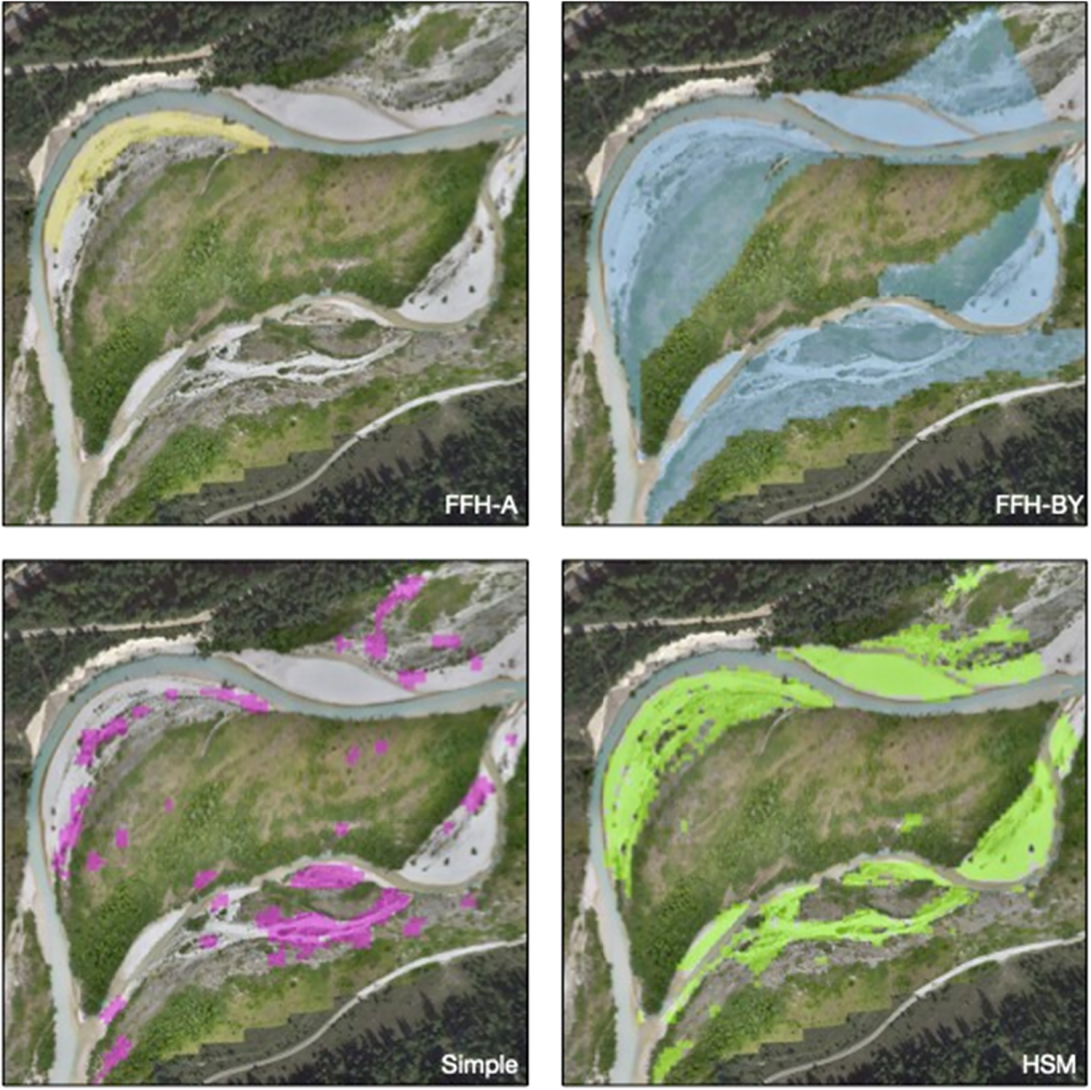
Delimitation of habitats suitable for *Myricaria germanica* based on the different mapping methods and models using the example of section W2. FFH-A: mapped according to Austrian requirements, focusing on minimum threshold values for *M. germanica* cover and maximum threshold values for *Salix ssp*. cover, FFH-BY: mapped according to Bavarian standards, solely based on the presence of *M. germanica* individuals, Simple: simple, intuitive Model solely based on vegetation cover and percent of sand in the surface substrate, HSM: habitat suitability model (gradient boosted regression) based on predictor variable vegetation cover, height above mean water level, percentage of sand in the surface substrate and vegetation height

The areas mapped as the habitat type “Alpine rivers with their ligneous vegetation of *M. germanica*” differ depending on whether the Bavarian or Austrian mapping guidelines are used. Similarly, there are notable discrepancies and a lack of consistent overlap between areas identified as suitable habitats for *M. germanica* by a simple model and the HSM (Fig. 2, Table 1).

**Table 1:** Total terrestrial area (in m²) of the respective river sections and the absolute and relative area mapped as habitat type by the respective guidelines and area identified as suitable habitat by simple and habitat suitability modeling

Section	Total area without water	BY without water^a^	A	Simple	HSM
M1	9600	9600 (100%)	2075 (22%)	1350 (14%)	2475 (26%)
M2	9275	9275 (100%)	1625 (18%)	3825 (41%)	5225 (56%)
W1	17125	17125 (100%)	2225 (13%)	7450 (44%)	11725 (68%)
W2	99825	69275 (70%)	4925 (5%)	11625 (12%)	31875 (32%)
V1	71275	67100 (94%)	0 (0%)	22850 (32%)	35925 (50%)
V2	172850	160350 (93%)	12100 (7%)	40725 (24%)	97350 (56%)
**total**	**379950**	**332725 (88%)**	**22950 (6%)**	**87825 (23%)**	**184575 (49%)**

^a^In accordance with the Bavarian mapping guidelines, the river course itself is included in the habitat delineation. To increase comparability, however, we excluded all water bodies from area assessments.

The area delineated using the Austrian guidelines accounts for only 6% of the study area, whereas the Bavarian guidelines map 88% of the area as the respective habitat type. Thus, the area defined as habitat type by the Bavarian guidelines is 14 times larger than that identified according to the Austrian guidelines. Furthermore, according to the Austrian guidelines, the proportion of area mapped as habitat type area per sample plot tends to decrease across the investigated river sections, which differ in their intensity of disturbance. In contrast, the proportion of habitat type area mapped according to the Bavarian guidelines remains relatively constant even though the sections differ in their level of degradation. This suggests that the Austrian method is more sensitive to differences in river dynamics, while the Bavarian method tends to result in more consistent outcomes, even when impairment conditions change. In many sections, the area mapped using the Bavarian guidelines encompasses the entire terrestrial area, including areas with dense vegetation in the herb and shrub layer (Table 1). Frequently occurring herb species in these areas are *Erica carnea, Sanguisorba minor, Prunella grandiflora, Potentilla erecta* and *Dryas octopetala*.

When comparing suitable habitat areas predicted by the simple model and the HSM, the HSM identifies a larger area as suitable for *M. germanica* germination and establishment. However, the proportion of suitable habitat varies across sample areas in both models. For the simple model, the proportion of suitable habitat within the respective sample areas ranges from 12% to 44%, while for the HSM, it ranges from 26% to 68%. Notably, in both models, the proportion of suitable habitat appears largely unaffected by variations in impairment intensity (Table 1).

Comparing habitat type areas delineated according to the Habitats Directive with modeled suitable habitat areas highlights further discrepancies (Table 2). When Austrian habitat mapping results are related to the HSM, which accurately predicts suitable habitats for germination and establishment of *M. germanica*, up to 52% of the modeled suitable area is either not classified as a habitat type or does not overlap with the classified areas. The majority of these sites are classified as false negatives, meaning that although the site is predicted to be suitable for *M. germanica*, it is not delineated as a habitat type due to insufficient *M. germanica* occurrence, indicating that not all suitable areas are occupied by *M. germanica*. This is confirmed by the varying occupancy of the species within suitable habitat predicted by the HSM across sections, ranging from 61% in Mittenwald to 13% in Vorderriss, with an average occupancy of 13% (Table 3). Conversely, the minor instances of false positive classifications according to the Austrian guidelines, meaning that M. germanica is relative abundant in areas classified as unsuitable by the HSM, fall within the model’s marginal error rate (3%). In contrast, a comparison of the Bavarian habitat type mapping results with the predicted suitable habitat reveals that the area classified as habitat type exceeds the suitable habitat by up to 74%, meaning that they overlap with areas considered as unsuitable for *M. germanica* germination and establishment. This confirms that the habitat type mapped according to Bavarian guidelines includes areas with dense herb and shrub vegetation in advanced stages of succession.

**Table 2:** Falsely evaluated (false positives/false negatives) are of suitable habitats by the different approaches, related to the results of the habitat suitability model as reference

Section	BY	A	Simple
M1	7125/0 (74%)	475/675 (12%)	50/1925 (21%)
M2	4050/0 (44%)	550/3800 (47%)	575/1950 (27%)
W1	5400/0 (31%)	300/8600 (52%)	1575/5050 (39%)
W2	37400/0 (37%)	500/26175 (27%)	3575/22700 (26%)
V1	31175/0 (48%)	0/35925 (50%)	8200/21025 (41%)
V2	63000/0 (36%)	950/84625 (50%)	5975/61675 (39%)

Percentage of totally falsely attributed area, shown for respective river sections. False positives mean an overestimation of suitable habitat area, and false negatives mean an underestimation. For more information on interpreting false positives and false negatives, see Chapter 2.2.4.

**Table 3:** Suitable habitat area and occupancy

Section	Area HSM	Occupancy compared to HSM	Occupied area %
M1	2475	1500	61%
M2	5225	1400	27%
W1	11725	2475	21%
W2	31875	5125	16%
V1	35925	300	1%
V2	97350	12625	13%
**Total**	**184575**	**23425**	**13%**

The predicted area as determined by the habitat suitability model (in m²), the actual occupied area, and occupancy (in %).

**Fig. 3:**
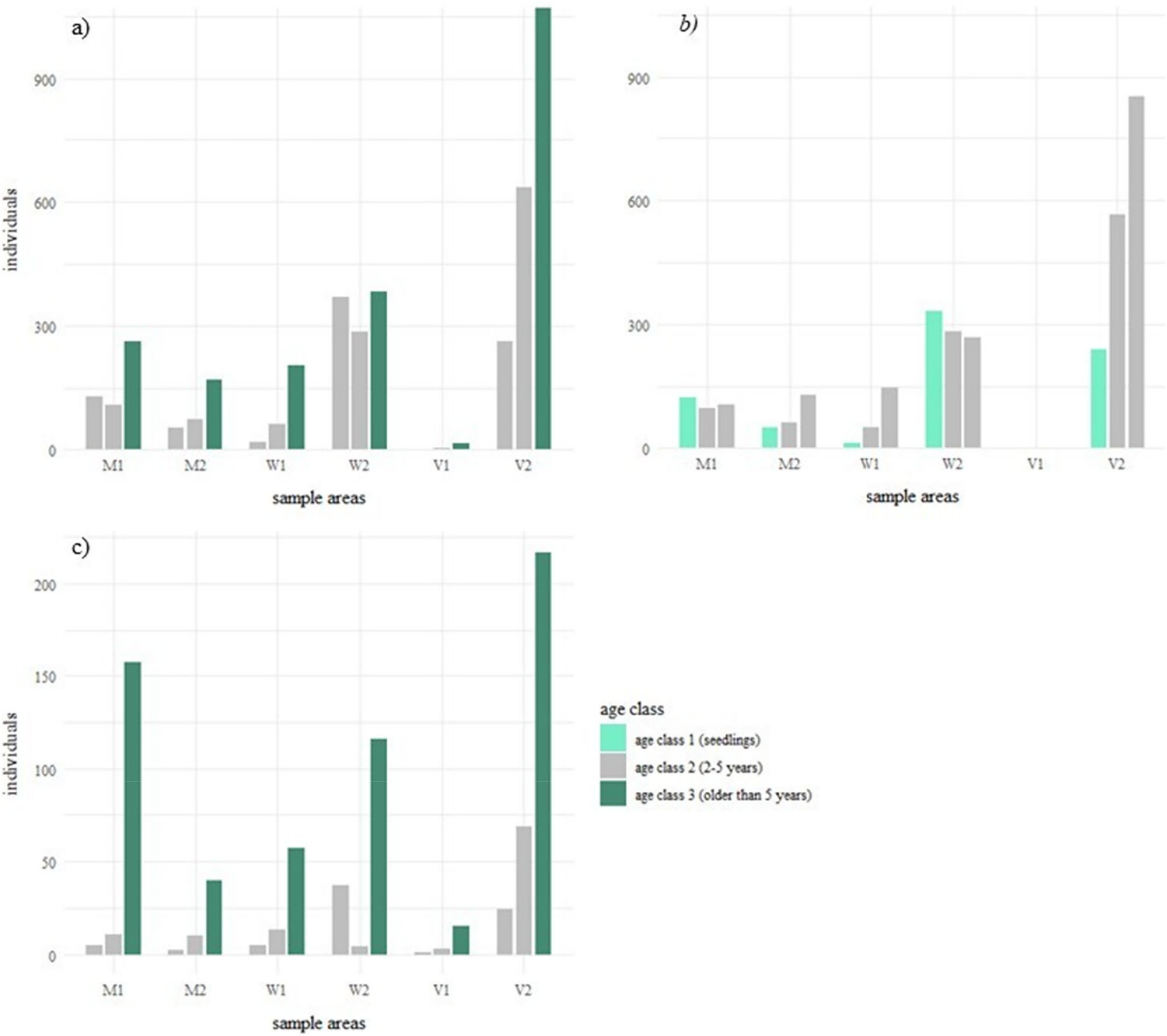
*M. germanica* individuals mapped according to the standards of the Habitats Directive across sample areas (M1–M2, W1–W2, V1–V2), categorized by age class: age class 1 (seedlings), age class 2 (2–5 years), and age class 3 (older than 5 years). **a** Individuals within the Bavarian delineation of the habitat type. As the Bavarian mapping guidelines classify any area with the presence of *M. germanica* as a habitat type, the Bavarian delineation covers all individuals occurring within the study area. **b** Individuals within the Austrian delineation of the habitat type. Due to defined thresholds—minimum *Myricaria* cover and maximum *Salix* shrub layer cover—not all individuals present in the study area are included in the habitat delineation under this method. **c** This is a subset of individuals that were recorded exclusively using the Bavarian method for habitat delineation. These are individuals that were not mapped using the Austrian approach and are thus not included in Austrian delineations of the habitat type

The age structure of the *M. germanica* population, recorded according to Bavarian standards, also shows that areas that are more advanced in succession are demarcated as habitat type: Individuals older than 5 years (age class 3) dominate the population. However, a comparison of the distribution of *M. germanica* within the habitat delineations of the Bavarian and Austrian guidelines shows that 786 individuals, representing almost one-fifth of the total population, are not covered by the Austrian guidelines. Notably, most of these unrecorded individuals belong to age class 3. In contrast, individuals less than five years old are predominantly located within the areas delineated by the Austrian guidelines. Thus, *M. germanica* populations within these designated habitats have a balanced age structure and ongoing rejuvenation. Individuals aged five years or younger (age class 1 and age class 2) are either predominant or balanced relative to older individuals (age class 3) (Fig. 3).

## Discussion

Braided riverscapes are in poor ecological status and continue to deteriorate within the EU and even globally (Grizzetti et al. 2017, Hohensinner et al. 2021, Islam et al. 2024). Halting further degradation and improving the ecological status of such riverscapes is both imperative and legally binding in the EU (European Commission 2000, 2024). Accurate monitoring of ecological conditions and habitat changes is, therefore, essential for effective conservation planning and ecosystem management.

As specified in the Interpretation Manual of European Union habitats, the Austrian and the Bavarian mapping method for “Alpine rivers with their ligneous vegetation of *M. germanica*” rely on the presence of *M. germanica* at the time of mapping (European Commission 2013, LWF & LFU 2018, Ellmauer et al. 2020). Given the species’ metapopulation dynamics, conservation requires monitoring methods that detect both shifts in habitat patches and changes in habitat availability (Kudrnovsky 2013, Lener et al. 2013, Wagner & Wöllner 2024).

The Bavarian mapping method applies broad delineation criteria, allowing even isolated *M. germanica* individuals to define the extent of the habitat type (LWF & LFU 2018). Consequently, entire river sections, including the watercourse and all gravel bars, are classified as habitat type. Potential germination sites of *M. germanica* are also delimited by this broad mapping approach. However, it often includes areas of extensive herb and shrub vegetation, often featuring a species composition that is atypical of the communities normally associated with the presence of *M. germanica*.

Moreover, a delineation method based solely on the presence of *M. germanica*, regardless of the plants’ age, life history stage, and cover, leads to the inclusion of areas in advanced stages of succession, as older individuals can persist for many years in higher successional stages (Egger et al. 2014). As a result, areas whose vegetation structure and composition are more accurately aligned with adjacent successional habitat types, are erroneously defined as ‘Alpine rivers with their ligneous vegetation of *M. germanica*’ solely due to the presence of a single *M. germanica* individual. This overlooks habitat decline caused by the aging of *M. germanica* stands and their succession into willow or grey alder scrubs, making a clear differentiation between the habitat types ‘Alpine rivers with their ligneous vegetation of *M. germanica*’ and ‘Alpine rivers and their ligneous vegetation with *Salix eleagnos*’ or ‘Alluvial forests with *Alnus glutinosa* and *Fraxinus excelsior* (*Alno-padion, Alnion incanae, Salicon albea*)’ unfeasible.

Therefore, the Bavarian mapping approach fails to detect site-specific changes driven by altered river dynamics, as its large-scale delineations mask small-scale changes. By encompassing entire river reaches, including vegetation-free gravel banks and active channels, the assessment of the “degree of conservation” is also distorted (Winkelhues 2024). For instance, the inclusion of non-vegetated surfaces leads to an underestimation of herb and shrub cover, key indicators for the assessment of habitat structures (LfU 2018, Winkelhues 2024). This “masking effect” has severe management implications: at the Upper Isar, for instance, the overly generous classification of the habitat type obscures the negative impacts of water diversion on river morphology. While the official management plan (Bayerische Forstverwaltung 2016) only identifies insufficient dynamics and *M. germanica* aging downstream of Untergries, numerous studies confirm these stressors are already evident directly below the Krüner Weir (Reich et al. 2008, Juszcyk et al. 2020, Reich & Rethschulte 2021, Maier et al. 2021). Consequently, habitat deterioration remains undetected until large-scale populations are entirely lost, effectively delaying essential restoration measures and complicating compliance with the monitoring requirements of the NRR. In contrast, the Austrian method delineates small-scale habitat patches where *M. germanica* is the dominant species (Ellmauer et al. 2020), effectively capturing shifts in local populations. The use of minimum cover thresholds for *M. germanica* is particularly valuable in detecting changes in habitat quality, such as progressive succession.

In highly dynamic ecosystems such as alpine braided rivers, habitat patches within the floodplain are constantly shifting (Kudrnovsky 2013, Arscott et al. 2002). The new, open habitats created by these dynamics are essential for *M. germanica* colonization, providing the pioneer sites needed for germination and establishment (Lener et al., 2013). As a species forming metapopulations in such dynamic environments, *M. germanica* often exhibits low occupancy rates and is frequently absent from areas that are otherwise suitable for its growth (Wagner & Wöllner 2024). This limitation poses a significant challenge for conservation, as protecting only individual or a small number of sites is insufficient for the long-term survival of *M. germanica* and its associated habitat (Kudrnovsky 2013, Lener et al. 2013, Wagner & Wöllner 2023). Thus, the long-term conservation of the *M. germanica* metapopulations depends not only on the protection of existing sub-populations. Antecedent habitats need to be protected as potential new habitat sites, as well as subsequent successional stages where older *M. germanica* individuals serve as diaspore dispersers (Winkelhues 2024).

These pre- and subsuccessional stages are also partially protected as EU habitat types. The habitat type “Alpine rivers and herbaceous vegetation along their banks” precedes in the successional sequence and roughly corresponds in its definition to the germination niche of *M. germanica* (Kudrnovsky 2013, Landmann 2013). A combined survey of this habitat type with the habitat type “Alpine rivers with their ligneous vegetation of *M. germanica*” would provide valuable insights into the long-term development potential of *M. germanica* habitats. Additionally, incorporating habitat type “Alpine rivers and their ligneous vegetation of *Salix eleagnos*” would provide information on regeneration and recolonization potential as well as a more comprehensive understanding of the overall state of the ecosystem. The feasibility of such comprehensive monitoring depends upon the resources available to Member States.

This is precisely where modeling approaches such as Habitat Suitability Modeling (HSM) can offer valuable support. As demonstrated by our results, HSM enables the realistic identification of potential habitats for *M. germanica* along river sections, while significantly reducing the need for extensive field surveys. Once created, the model can be saved and used applied to other rivers and other times, enabling time series and realistic comparison without field mapping. It can thus serve as a useful complementary tool, for example, in the development of management plans. However, HSM should be viewed strictly as a supplement to field-based assessments required under the Habitats Directive. As modeling helps to delineate the habitat potential. While modeling is well-suited for delineating potential habitat, the identification of actually occupied, or realized, habitat patches requires consistent, cross-national field mapping and assessment methodologies. Ultimately, both realized habitats and areas with colonization potential must be protected to ensure the long-term persistence of *M. germanica* and its dynamic habitat.

A best-practice example for mapping dynamic habitat types is the Lower Saxony method for dune habitat types (Drachenfels 2012). It evaluates the degree of conservation by considering the full range of dune habitat types, acknowledging that natural dynamics create new pioneer stages while older ones persist elsewhere. If all successional stages are sufficiently represented, the whole complex is assessed as in a good condition (Drachenfels 2012). Applied to alpine braided rivers, the degree of conservation would accordingly be assessed by a combined evaluation of the entire succession sequence, encompassing the habitat types 3220, 3230, 3240, 91E0, and possibly 6430. Such an inventory would provide a more comprehensive picture of the degree of conservation and thus the dynamics of alpine braided rivers.

One of the central aims of the Habitats Directive, as part of the Natura 2000 network, is to establish a coherent European ecological network (European Commission 1992). Thus, striving for a uniformity of implementation of the Directive across Member States. However, in practice, mapping and assessment methods for the habitat type “Alpine rivers and their ligneous vegetation of *M. germanica*” vary significantly, leading to inconsistent evaluations of the same situation. Not only do guidelines for mapping and assessing the degree of conservation of this habitat type, and its adjacent habitat types, differ (Winkelhues 2024), but the threshold values used to determine conservation status also vary across the EU for all habitat types (Ellwanger et al. 2018). Furthermore, Member States show considerable differences in both their approach and progress in implementing the Habitats Directive (Ellwanger et al. 2018, Genovart et al. 2025). The inconsistencies in implementing the Habitats Directive, as highlighted by our analysis, make it difficult to evaluate the conservation status of habitats across the EU. This is particularly evident for habitat type 3230, which occurs across ten EU Member States and three biogeographic regions (Alpine, Continental, and Mediterranean) (EEA 2025). Given that rivers vary considerably in altitude, size, flow regime, and habitat structure across these regions (Kudrnovsky 2013), diverging national mapping approaches are likely to introduce substantial bias into EU-wide assessments of conservation status. A comprehensive comparison of mapping methodologies across all Member States and biogeographic regions where habitat type 3230 occurs would therefore be a valuable avenue for future research. Beyond that, Genovart et al. (2025) report comparable challenges in species monitoring, where widespread non-compliance with standardized EU monitoring guidelines has resulted in a fragmented data landscape, preventing reliable comparisons of conservation status. In the case of alpine braided river habitat types, inconsistencies in the implementation of the Habitats Directive are particularly relevant for assessing the indicator “structure and functions”, which includes evaluations of the “degree of conservation” of habitat patches. These inconsistencies not only complicate compliance with the non-deterioration principle—a core aspect of the Habitats Directive (European Commission 1992) - but may also hinder the effective implementation of the NRR, which requires measuring improvements in habitat conditions and restoration of free-flowing rivers. Although the monitoring requirements of the NRR are not exactly the same as those of the Habitats Directive, there is overlap as the areas protected under the Habitats Directive form the backbone of the NRR (European Commission 2024). Halting further degradation or improving the ecological status of alpine braided rivers, one of Europe’s most endangered ecosystems, remains a significant challenge. Although the Natura 2000 and the NRR aim to move beyond fragmented, “conservation up to the border” approaches, their practical implementation varies, and cross-border coordination can be limited. This issue is particularly critical for longitudinal ecosystems such as (braided) rivers that often extend across administrative and national boundaries.

In our case study of the Upper Isar River, conservation, monitoring, and management are conducted separately in Germany and Austria, even though the Isar and several of its tributaries originate in Austria. An even more extreme example is the Lech River: while Austria has implemented extensive conservation and restoration efforts upstream, the river is heavily regulated on the German side (Egger et al. 2019). As a result, species and habitat types that are protected and supported in Austria lose their habitat just 15 kilometers downstream in Germany.

A more unified, cross-border implementation of the Habitats Directive, particularly in the mapping and assessment of braided river habitat types, is urgently needed. However, the feasibility of such standardization remains uncertain, as establishing uniform monitoring programs for all Member States would require substantial coordination efforts (Ellwanger et al. 2018). Furthermore, Borras et al. (2014) emphasize that the progress toward standardization is also hindered by ambiguities within the directive itself, as well as by the complex spatial and temporal interactions among local administrative authorities and stakeholders. Moreover, for the investigated habitat type, “Alpine rivers with their ligneous vegetation of *M. germanica*,” habitat characteristics vary across sites due to differences in altitude, runoff behavior, and geological conditions (Kudrnovsky 2013). Despite these variations, we strongly recommend standardizing mapping and assessment guidelines for the “degree of conservation” to a level that ensures the ecological characteristics of habitat types, as defined in the Interpretation Manual, are accurately represented. For a more consistent mapping of “Alpine rivers with their ligneous vegetation of *M. germanica*,” methods and guidelines should at least be standardized in terms of delineation and the consideration of *M. germanica*’s population structure when assessing the “degree of conservation”.

## Conclusion

Ecosystems and their biodiversity transcend international or administrative boundaries, necessitating conservation efforts extending beyond national borders. This is particularly crucial for braided rivers, which rely on dynamic processes. Our study highlights that even the Habitats Directive – intended to set up an EU-wide nature conservation network - partially fails to ensure effective cross-border nature protection of braided river habitat types. Although the directive provides a joint administrative framework, habitat assessments and degree of conservation evaluations vary between Member States. These discrepancies can lead to significantly different assessments of the situation, and potentially different management and conservation strategies, depending on which Member State-specific method is used. Our study thereby emphasizes the urgent need for a standardized, Europe-wide mapping and assessment procedure that accounts for the characteristics of dynamic riverscapes while accurately capturing small-scale and short-term changes in the habitat situation. To achieve this, we recommend that the habitat type should be delineated based on the actual presence of *M. germanica*, defined by a minimum coverage threshold of >1%. To ensure that no potential germination niches are overlooked, we recommend surveying the habitat type alongside its successional predecessors and successors (i.e., habitat type 3220 and 3240) without conflating the boundaries of these distinct habitat types. Such a combined survey would provide a more comprehensive assessment of the long-term development potential of *M. germanica* habitats and the overall dynamic state of the ecosystem. Additionally, stronger cross-border cooperation is essential to ensure consistent conservation outcomes across the EU.

Standardized habitat mapping and assessment will also be critical for implementing the NRR, which requires Member States to monitor the progress and success of habitat restoration. The NRR is especially relevant for braided rivers, as it mandates the restoration of 25,000 kilometers of free-flowing rivers across Europe (Van De Bund et al. 2024). The success of these restoration efforts may be evaluated based on the presence of habitat types characteristic of such river systems.

## Supplementary information

Supplementary material

## Data Availability

The datasets generated during and/or analyzed during the current study are available from the corresponding author on reasonable request.
