# Supplementary material for: Bridging Conservation Gaps: Evaluating Habitat Mapping Methods for Alpine River Ecosystems
Source: Environ Manage. 2026 Jun 21;76(7):221. doi: 10.1007/s00267-026-02527-9 (PMC13284007; doi:10.1007/s00267-026-02527-9)
Supplement: Supplementary file 1 — Supplementary material [file 267_2026_2527_MOESM1_ESM.docx]

**Bridging Conservation Gaps: Evaluating Habitat Mapping Methods for Alpine River Ecosystems**

Wiebke Winkelhues^a^, Thomas C. Wagner, Helmut Kudrnovsky, Carmen Rethschulte,
Michael Reich

^a^ Institute of Environmental Planning, Leibniz University of Hannover, Herrenhäuser Straße 2, 30419 Hannover, Germany
E-mail: winkelhues@umwelt.uni-hannover.de

**Supplementary Information (SI)**

**Supplementary 1 (S1) Degradation, interventions and impact, daily mean and mean maximum discharges for the three river sections of the River Isar.** Discharge measurements were taken from the nearest river gauge stations “Mittenwald” (ID 16000708) and “Rissbachdüker” (ID 16001303); data of the respective stations was obtained from the Bavarian State Institute of the Environment (LfU Gewässerkundlicher Dienst, 2025)

| **Section** | **Degradation** | **Interventions and impacts** | **MQ [m³]** | **MHQ [m³]** | **Sample  Area** | **Date of recording** |
| --- | --- | --- | --- | --- | --- | --- |
| Mittenwald | Semi-natural | Upstream straightening of the Isar for Mittenwald flood protection, but natural and morphological dynamics persist | 16.9 | 58.4 | M1 | May 2023 |
|  |  |  |  |  | M2 | May 2023 |
| Wallgau | Semi-natural | Krüner weir diverts up to 25 m³/s leaving a minimum residual flow of 3–4.8 m³/s. Reduced residual flow decreases velocity, transport capacity, bedload, and flood peaks. | 6.05 | 104 | W1 | May 2023 |
|  |  |  |  |  | W2 | June 2023 |
| Vorderriß | Near-natural | Large braided floodplain with near-natural sediment dynamics; bedload deficit from Krüner weir largely compensated by the Rissbach during floods | 6.05 | 104 | V1 | June 2023 |
|  |  |  |  |  | V2 | June 2023 |

**Supplementary 2 (S2)** **Validation of the habitats predicted by the habitat suitability model** against the presence of young *Myricaria germanica* within the respective sample areas

| **Sample area** | ***M. germanica***  **age class <2 a** | **Individuals within  the predicted area** | **Proportion**  **correctly predicted** |
| --- | --- | --- | --- |
| M1 | 128 | 106 | 83% |
| M2 | 52 | 42 | 81% |
| W1 | 344 | 338 | 98% |
| W2 | 21 | 20 | 95% |
| V1 | 1 | 1 | 100% |
| V2 | 261 | 251 | 96% |
| **Total** | **807** | **758** | **94%** |

*
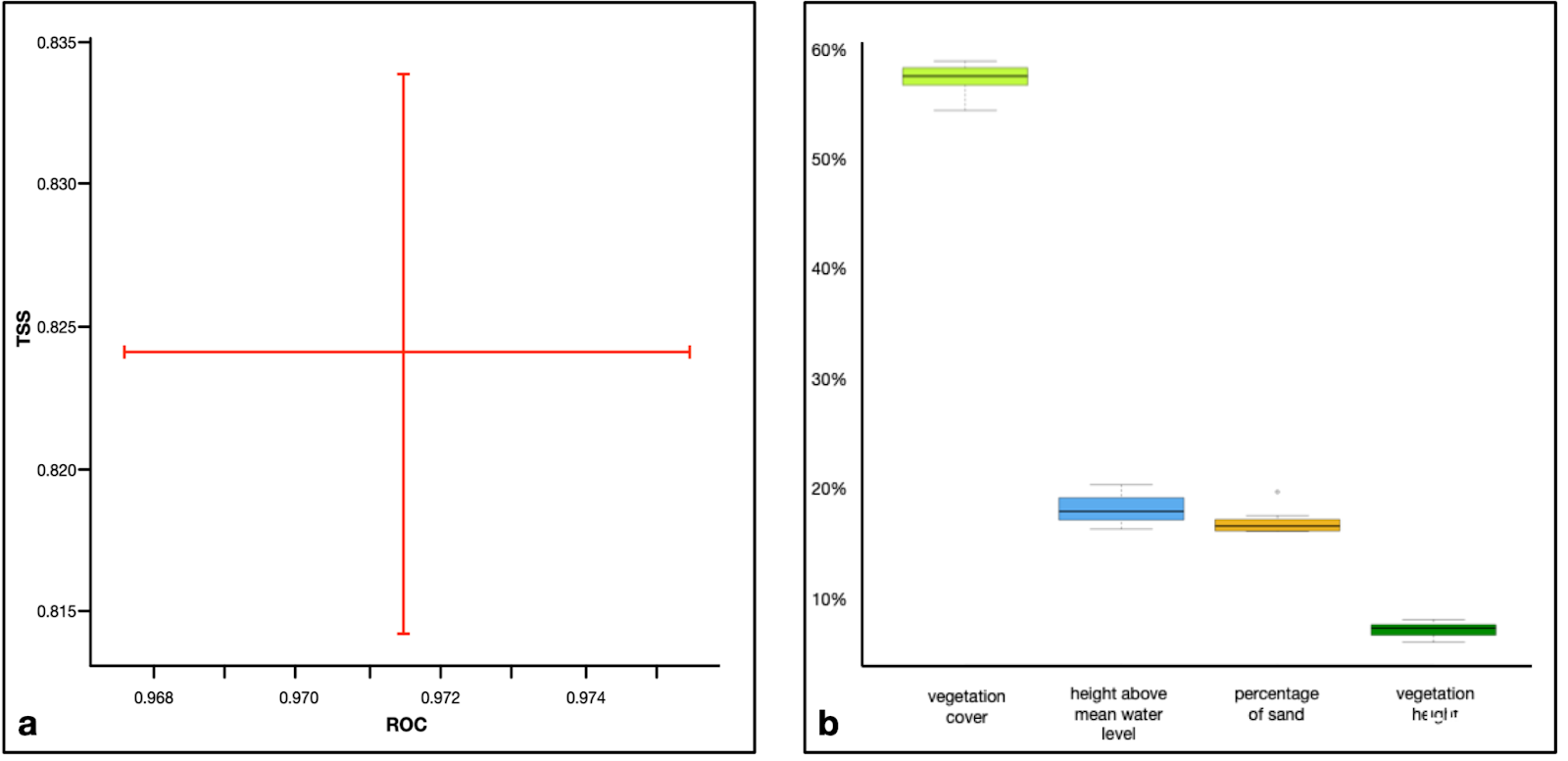
*

**Supplementary 3 (S3)** **HSM diagnosis.** ROC/TSS values a) and relative variable importance b) of the ten submodels
